# EHEALTH INTERVENTIONS TO PROMOTE PHYSICAL ACTIVITY AMONG OLDER ADULTS WITH MCI: A SYSTEMATIC REVIEW

**DOI:** 10.1093/geroni/igae098.2449

**Published:** 2024-12-31

**Authors:** Jinhee Shin, Kennedy Diema Konlan, Chang Gi Park

**Affiliations:** Woosuk university, Jeollabuk-do, Ch’ungch’ong-bukto, Republic of Korea; University of Health and Allied Sciences, Ho, Volta, Ghana; University of Illinois Chicago, Chicago, Illinois, United States

## Abstract

Physical activity positively influences and improves motor ability, increases desirable behavior, promotes brain activity, and increases marginal memory and thinking among older adults with mild cognitive impairments. This systematic review explored current trends and the effectiveness of digital health (eHealth) interventions to promote physical activity among older adults with mild cognitive impairment. We used the PRISMA guidelines and the PICO framework to search for literature in five electronic databases (PubMed, Embase, CINAHL, and Web of Sciences). The search outcome was transferred to Endnotes software and independently screened based on title, abstract, and full text using predetermined eligibility criteria. The screening resulted in 10 articles for extraction using a self-developed matrix. The JBI quality appraisal tool was used for quality appraisals and the conventional thematic data synthesis method of analysis was used. The strategy used was diverse and multiple technological platforms for the implementation of digital interventions, and included hardware technology like tablets, smartphones, smartwatches, Bluetooth devices, earpieces, computers, or durable covers, while, software techniques like promoting, customization, multimedia, multisensory, hands-on practice, demonstration, cueing, tech support, user manual or in the training of care practitioners were used. The use of digital-based health interventions for people with mild cognitive impairment to improve physical activity is still rudimentary even though the potential influence of such interventions is enormous. Interventions that use digital methods focusing on promoting physical activity among older adults with mild cognitive impairments should provide detailed information on the types of interventions, implementation, development process, and the resultant improvements.

